# Low Rate of Postoperative Pterygium Recurrence in Patients under Treatment with Low-Dose Oral Doxycycline for Chronic Blepharitis: A First Report

**DOI:** 10.3390/diagnostics14070715

**Published:** 2024-03-28

**Authors:** Fiammetta Catania, Paolo Vinciguerra, Alessandra Di Maria

**Affiliations:** 1 Department of Biomedical Sciences, Humanitas University, Via Rita Levi Montalcini 4, 20072 Pieve Emanuele, Italy; paolo.vinciguerra@humanitas.it; 2 IRCCS Humanitas Research Hospital, via Manzoni 56, 20089 Rozzano, Italy; alessandra.di_maria@humanitas.it

**Keywords:** pterygium, recurrence, doxycycline, ocular surface inflammation, metalloproteases

## Abstract

Purpose: Low doses of systemic doxycycline (LD-SD) inhibit angiogenesis and the expression of matrix metalloproteases, which are determinants of pterygium progression. This study aimed to compare the recurrence rate and visual outcome of pterygium excision in patients undergoing chronic treatment with LD-SD for chronic refractory blepharitis and LD-SD-naive patients. Methods: A retrospective analysis of patients that underwent surgical excision and conjunctival graft apposition was conducted. Patients were divided in a TETRA group (under LD-SD treatment at the moment of surgery) and a control group. The main outcome was the rate of recurrence at 1 year postoperatively. Secondary outcomes were the comparisons of surface regularity, visual quality, and dry-eye symptoms at 6-week, 6-month, and 1-year follow-up in the two groups. Results: The TETRA group showed a significantly lower rate of 1-year recurrence both in primary (*p* = 0.034) and recurrent (*p* < 0.001) pterygia. The best corrected visual acuity (BCVA), astigmatic error, corneal total root mean square (RMS), and ocular surface disease index (OSDI) significantly reduced during the follow-up in both groups. The surface asymmetry index and high-order aberrations (HOAs) significantly reduced only in the TETRA group. The final BCVA was significantly higher, while the OSDI score and total RMS and HOAs were significantly lower in the TETRA group compared to the control. Conclusions: Patients under treatment with LD-SD showed a lower rate of recurrence at 1-year follow-up compared to controls. These patients also experienced higher BCVA and surface regularity and less dry-eye symptoms.

## 1. Introduction

Clinically significant pterygium induces poor visual quality due to tear film alteration, the induction of astigmatism, media opacity, photophobia, epiphora, and binocular diplopia due to the contraction of Tenon’s capsule [1]. Surgical excision is the primary treatment, even though 65–88% of recurrences within the first postoperative year are reported in cases where no preventive method is applied [2]. This event is particularly undesirable, since recurrences and repeated surgeries complicate the clinical outcome, as the growth of the lesion may become more aggressive and cause high-order aberrations (HOAs) [3]. For this reason, in the past few decades, many strategies have been attempted to reduce the incidence of recurrences, including surgical techniques such as modified bare sclera techniques with the transposition of the conjunctival flap [4,5], conjunctival autograft transplantation [6], amniotic membrane transplantation [7], and peripheral lamellar keratoplasty (in cases of significant ingrowth) [8]. The effectiveness of topical adjuvant therapy has also been evaluated, which include techniques such as mitomycin C [9] and 5-fluoruracil [10] application on the pterygium bed and intraoperative subconjunctival injection [11,12] or postoperative eye-drop application [13,14] of anti-VEGF. The use of conjunctival or limbal autografts and/or the use of topical mitomycin C (MMC) after pterygium excision have been demonstrated to reduce the recurrence rate compared with bare sclera excision alone, and they are currently the most adopted techniques [15,16]. Even though the outcomes of the two techniques are very similar, topical MMC has resulted in a significantly higher amount of intra- and postoperative complications [17,18]. Also, amniotic membrane graft has been demonstrated to be less effective in preventing recurrence compared to conjunctival autograft, with a recurrence rate ranging from 6.4% to 42% [19]. In fact, conjunctival autograft apposition or transposition has been demonstrated to reduce the recurrence risk to 5–15% [2], but higher rates have been reported in larger or recurrent pterygia [20,21]. As a consequence, new methods that could further reduce the incidence of recurrence are particularly awaited. To this purpose, low doses of systemic doxycycline (LD-SD) have been proven to inhibit both the expression and activation of stromal cells’ matrix metalloproteases 2 (MMP-2) and MMP-9, whose expression is particularly relevant to pterygium progression [22,23,24]. Furthermore, low-dose doxycycline produces antiangiogenic effects through the inhibition of vascular endothelial growth factor and the migration of smooth-muscle cells, which are important pathogenetic events in pterygium invasive processes [25,26,27,28,29,30]. The use of LD-SD has recently been proposed as a treatment for chronic refractory blepharitis, a condition that shows a significant clinical overlap with pterygium, conferring a two-fold risk of incidence in affected patients [31,32,33,34]. In particular, the treatment is indicated by the American Academy of Ophthalmology guidelines [35] in people with posterior blepharitis or symptoms not adequately controlled by lid hygiene and topical medications, especially those with concurrent meibomian gland dysfunction (MGD). The recommended dosage is 50 mg twice a day (100 mg per day) for the first two weeks and a chronic maintenance therapy of 50 mg per day [36]. Clinical improvement requires several weeks, but once it is achieved, therapy may be discontinued or tapered to maintenance doses [37]. The aim of the present study is to assess the 1-year rate of recurrence of both primary and recurrent clinically significant pterygium after surgical excision and conjunctival graft apposition in a cohort of patients undergoing chronic treatment with LD-SD for chronic refractory blepharitis and to compare it to patients naïve to LD-SD. Moreover, we wish to compare changes in best corrected visual acuity (BCVA), topographical surface regularity, and dry-eye symptoms during the first postoperative year within the two populations. Lastly, the fact that this article’s rationale stands on experimental evidence of the inhibitory action of doxycycline on metalloproteases and ocular surface inflammation and aims to find applications for this in the prevention of pterygium recurrence after surgery confers translational relevance to the topic.

## 2. Materials and Methods

### 2.1. Study Design and Patient Selection

This study retrospectively analyzed patients referred to the Ophthalmology Department of the Humanitas Clinical and Research Center (Rozzano, Italy) from June 2019 to December 2021 for the surgical removal of pterygium. All included patients were affected by chronic refractory blepharitis (defined as blepharitis resistant to treatment with lid hygiene and topical medication after at least 6 months of good-compliance treatment). Blepharitis was diagnosed based on a slit lap examination in the presence of anterior (redness and crusting of the anterior margin) or posterior (Meibomian gland cupping and obstruction) lid inflammation. Moreover, all of them showed a pterygium involving at least 5 rings at preoperative keratoscopy. Cases of both primary (n = 69) and recurrent pterygium (n = 43) were included. Only patients with available high-quality topography acquisitions were included, defined by a >90% centration and a >80% coverage of the acquisition. All patients underwent excision of the lesion and conjunctival autograft transplantation or transposition. Only one study eye was randomly selected for each patient in case of bilateral disease. The exclusion criteria were other corneal or scleral diseases (with the exception of dry-eye disease and blepharitis), the preoperative and postoperative use of any kind of topical eyedrops with the exception of chronic use of artificial tears and routine postoperative corticosteroid and antibiotic treatment, an absence of data assessing the presence of a recurrence at 1 year, and intraoperative complications. Patients that had been under treatment with LD-SD for at least 4 weeks at the moment of surgery and that continued treatment for at least 6 weeks after surgery were included in the TETRA group, while naïve patients were included in the control group. 

#### Objectives of This Study and Outcome Measures

Primary objective of this study was to describe the rate of corneal recurrence of primary and recurrent pterygium after surgery in the study population. Recurrence was defined as the development of any recurrent pterygium located on the primary surgical site greater than 1 mm in size anterior to the limbus [38]. Recurrence was assessed with an anterior segment slit lamp examination and/or keratoscopy during regular follow-up visits. Secondary objectives of this study were to describe surface regularity, visual quality, and ocular symptoms (focusing in particular on dry-eye symptoms) over time in the study population. Surface regularity was determined by analyzing the variation in the following topographic indexes: the surface asymmetry index (SAI) [39], longitudinal spherical aberration (LSA) [40], and the symmetry index of curvature (IC) [41].

Visual quality was assessed by analyzing the variation in BCVA, spherical and cylindrical subjective refractive error expressed in diopters, and optical aberrations over time. A MODI 2 CSO topographer was used to acquire data for both topographic and aberrometric analysis. The corneal total root mean square (RMS) and cornea-derived high-order aberrations (HOAs) were considered as outcome measures. Ocular symptoms were evaluated by registering reported symptoms at every follow-up and collecting data from the ocular surface disease index (OSDI) administered for the monitoring of blepharitis.

### 2.2. Procedures

**Figure 1:**
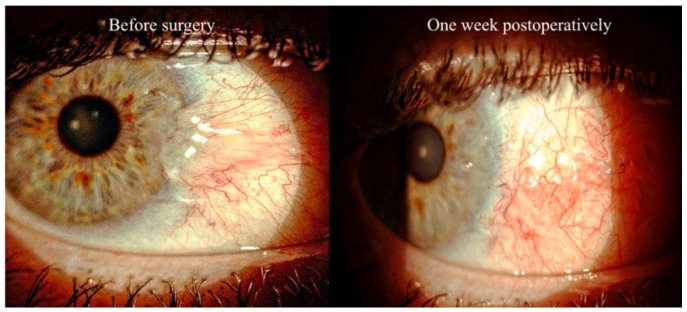
Anterior segment photos before (**left side**) and one week after (**right side**) surgical excision with superior conjunctival transposition in a patient with primary pterygium.

All operations were performed by the same surgeon (A.D.M.). Surgical removal of the pterygium was performed under subconjunctival anesthesia with 2% lidocaine and topical novesine eyedrops. The pterygium was detached from the invaded cornea with a Crescent blade and the pathologic tissue was removed along with ipsilateral conjunctiva. The bare sclera was then covered with either a free conjunctival autograft flap harvested from the superior quadrant or a conjunctival transposition from the superior quadrant. The conjunctiva was then sutured with 8.0 re-absorbable polyglactin 910 (Figure 1). At the end of the treatment, the patient was medicated with ofloxacin ointment. Postoperative treatment included applications of ofloxacin ointment 3 times per day for 5 days, as well as an ophthalmic gel containing sodium hyaluronate, xanthan gum, and netilmicin 3 times a day for 10 days. Patients were also treated with prednisolone acetate every 2 h for 3 weeks and then four times daily for a further 6 weeks [42]. They all received chronic postoperative treatment for blepharitis with artificial tears and lid hygiene.

Follow-up data from the first postoperative year were collected, focusing on 3 timepoints: 1 month, 6 months, and 1 year after the surgery. The Humanitas Clinical and Research Center Ethics Committee ruled that, on this basis and according to the Italian law, formal approval was not required for this monocentric retrospective study. This report of protocol awareness, conducted according to the ethical standards set in the 1964 Declaration of Helsinki, as revised in 2000, was deemed sufficient. All the authors reviewed the manuscript and vouched for the accuracy and completeness of the data and for the adherence of this study to the protocol.

### 2.3. Statistical Analysis

Statistical analysis was conducted using the SPSS software (IBM SPSS Statistics 26.0). Sample size calculation was performed considering a 5% alpha error, a 20% beta error, and a mean recurrence rate for pterygium excision with conjunctival graft apposition of 10% (range between 5 and 15%) [2]. The normality of the distribution for quantitative variables was evaluated using the Shapiro–Wilk test. Normally distributed variables were described using mean and standard deviation. Qualitative variables were described as the number of cases out of the total and percentages. A one-way ANCOVA for repeated measures was used to assess variations over the course of the follow-up in quantitative outcome measures. A two-way ANOVA for repeated measures was used for the comparison of changes in quantitative variables between the study populations. Qualitative differences between the two populations were assessed with Chi square test. Post hoc analysis was performed to assess individual differences. A *p* value < 0.05 was considered as statistically significant.

## 3. Results

### 3.1. Tetra Group: Descriptive Analysis

The TETRA group included 36 eyes with primary pterygium and 24 eyes with recurrent pterygium. The treatment protocol for all patients was the standard protocol adopted for refractory blepharitis treatment (a single dose of a 50 mg doxycycline pill in the morning at least 30 min before breakfast and accompanied by a glass of water). No patient experienced a decrease in corneal transparency after surgery.

#### 3.1.1. Primary Pterygium

The mean age of patients with primary pterygium was 45.69 ± 14.27 years, and 44.4% of patients (16/36) were of male sex. In this group, 61.1% were of Caucasian ethnicity (22/36), while the remaining 38.9% were of Hispanic ethnicity. The mean duration of the treatment with LD-SD was 4.7 ± 1.3 months before surgery and 7.2 ± 3.8 months after surgery. Nineteen out of thirty-six patients (52.7%) remained under LD-SD at least 6 months after surgery, while 3 of 36 (8.3%) were still under treatment with LD-SD 1 year after surgery. Sixteen patients (44.4%) reported more than 5 h per day of sun exposure and the disease involved the right eye in 50% of the cases (18/36) (see Table 1). Only 1 patient (aged 55) out of the 36 (2.77%) developed recurrence at 1-year follow-up. The mean BCVA at baseline was 0.79 ± 0.11 decimals, but it increased to 0.92 ± 0.06 decimals after 6 weeks from surgery and to 0.96 ± 0.038 decimals at 6-month follow-up (*p* = 0.037). Moreover, a significant decrease in astigmatic refractive error was detected between baseline and 6-week follow-up (*p* = 0.021). As concerns topographic data, the SAI was significantly lower at the 6-week examination than at baseline (*p* = 0.042) and continued to decrease over the entire course of the follow-up (see Table 1). Similarly, the corneal total RMS was significantly reduced at the end of the 1-year follow-up compared to the baseline assessment (*p* = 0.039), while the reduction in HOAs showed a trend towards statistical significance (*p* = 0.058) (Table 1). The mean OSDI score at baseline was 26.85 ± 8.42, which was significantly higher than the 6-week score of 14.07 ± 6.15 (*p* = 0.025). 

#### 3.1.2. Recurrent Pterygium

The mean age of patients with recurrent pterygium was 50.31 ± 9.62 years, and 41.67% of patients (10/24) were of male sex. The majority of patients were of Hispanic ethnicity (83.33%). The mean number of previous surgeries was 2.3 ± 1.1. The first excision surgery was the bare sclera technique in 12/24 cases (50.0%), excision and conjunctival graft apposition in 7/24 cases (29.2%), and excision and amniotic membrane in 5/24 (20.8%) cases. The mean duration of the therapy with LD-SD was 5.5 ± 1.1 months before surgery and 8.0 ± 3.6 months after surgery. Fourteen out of twenty-four patients (58.3%) received at least 6 months of postoperative treatment with LD-SD, while 3 out of 24 were still under treatment with LD-SD after 1 year.

Sun exposure for more than 5 h per day was reported in 4/24 (16.67%), and the right eye was involved in 58.33% of cases (see Table 1). Clinical recurrence was detected in 1 patient (aged 61) out of the 24 (4.16%) at the 1-year visit. The mean BCVA at baseline was 0.56 ± 0.19 decimals, with a mean cylindrical refractive error of −2.53 ± 0.83 D. The BCVA significantly increased (*p* < 0.001) and the astigmatic refractive error significantly decreased (*p* = 0.002) at the 6-week examination, and they continued with a similar trend over the course of the follow-up. The mean SAI at baseline was 1.31 ± 0.82. Patients with recurrent pterygium experienced a decrease in SAI (*p* = 0.004), LSA (*p* = 0.021), and IC (*p* = 0.043) over the course of the follow-up (see Table 1). Notably, the corneal total RMS and HOAs also significantly decreased during the first postoperative year (respectively, *p* = 0.036 and *p* = 0.041). The mean OSDI score at baseline was 24.0 ± 8.91, which was statistically significantly higher than the follow-up score (*p* = 0.005). The highest difference was registered between the baseline and 6-week measurements for all mentioned variables (see Table 1). 

### 3.2. Control Group: Descriptive Analysis

The control group included 33 eyes with primary pterygium and 19 eyes with recurrent pterygium. No patient experienced a decrease in corneal transparency after surgery.

#### 3.2.1. Primary Pterygium 

The mean age of patients with primary pterygium was 41.39 ± 13.17 years, and 45.5% of patients (15/33) were of male sex. In this group, 63.6% were of Caucasian ethnicity (21/33), while the remaining 36.4% were of Hispanic ethnicity. Thirteen patients (39.4%) reported more than 5 h per day of sun exposure, and the disease was involved the right eye in 48.5% of cases (16/33) (see Table 2). Three patients out of the thirty-three (9.1%) recurred at 1-year follow-up (aged 47, 51, and 53). The mean BCVA at baseline was 0.75 ± 0.10 decimals and increased to 0.81 ± 0.09 decimals 6 weeks after surgery and to 0.86 ± 0.04 decimals at 6-month follow-up (*p* = 0.045). No significant changes in spherical refractive error were reported (*p* = 0.463), while a significant decrease in astigmatic refractive error was detected between the baseline and 6-month follow-up (*p* = 0.028). As concerns topographical data, the SAI, LSA, and IC did not change significantly over the course of the follow-up (respectively, *p* = 0.561, *p* = 0.499, and *p* = 0.672). The corneal total RMS was significantly reduced at the end of the 1-year follow-up compared to the baseline assessment (*p* = 0.046), while HOAs did not significantly decrease over the first postoperative year (*p* = 0.112) (Table 2). The mean OSDI score at baseline was 25.32 ± 6.81 and significantly decreased at 1-year follow-up to 17.11 ± 5.26 (*p* = 0.046). 

#### 3.2.2. Recurrent Pterygium

The mean age of patients with recurrent pterygium was 52.41 ± 7.11 years, and 42.11% of patients (8/19) were of male sex. The mean number of previous surgeries was 2.1 ± 1.5. The first excision surgery was the bare sclera technique in 9/19 cases (47.4%), excision and conjunctival graft apposition in 5/19 cases (26.3%), and excision and amniotic membrane in 5/19 (26.3%) cases. The Hispanic ethnicity characterized 15 out of 19 patients (78.9%), while sun exposure for more than 5 h per day was reported in 4/19 (21.05%). Three patients out of nineteen manifested clinical recurrence at 1-year follow-up (aged 55, 59, and 48). The mean BCVA passed from 0.59 ± 0.13 decimals at baseline to 0.81 ± 0.09 decimals at 1-year follow-up (*p* = 0.027). Spherical refractive error did not change significantly (*p* = 0.483), while the cylinder decreased from −2.27 ± 1.03 D at baseline to −1.35 ± 0.33 D at 1 year (*p* = 0.029). No significant changes in SAI (*p* = 0.516), LSA (*p* = 0.213), and IC (*p* = 0.395) were noted over the course of the follow-up (see Table 2). Similarly, no significant changes in HOAs were present (*p* = 0.225). By contrast, the total corneal RMS significantly decreased at 1 year (*p* = 0.041). The OSDI score deceased from 27.33 ± 6.11 to 20.93 ± 5.83 at 1 year, but this result was only borderline in terms of statistical significance (*p* = 0.072) (see Table 2).

### 3.3. Comparison between Groups 

A comparison of the relevant characteristics other than outcome measures concerning the preoperative and postoperative status of the eye in the two study groups is summarized in Table 3. Preoperative DED was defined as the presence of either a pathological Schirmer test (<10 mm in 5 min) or a pathological tear break up time (<10 s) despite treatment with LD-SD.

#### 3.3.1. Primary Pterygium

There were no significant differences in terms of age (*p* = 0.164), sex composition (*p* = 0.882), ethnicity (*p* = 0.568), sun exposure (*p* = 0.109), and laterality (*p* = 0.411) between patients with primary pterygium in the TETRA and control groups. Moreover, no significant differences in terms of BCVA, spherical refractive error, cylindrical refractive error, LSA, IC, SAI, corneal total RMS, and OSDI score were detected between the two groups at baseline (*p* < 0.05). The rate of recurrence was significantly lower in the TETRA group (*p* = 0.034). In fact, 1/24 eyes (2.8%) in the TETRA group developed a recurrence (1.3 mm) between the 6-month and 1-year follow-up visits. In the control group, 3/33 (9.1%) eyes developed a recurrence during the first postoperative year: one was detected at the 1-year follow-up timepoint (1.5 mm), while the other two were detected at the 6-month follow-up timepoint (2.3 mm and 1.2 mm).

**Figure 2:**
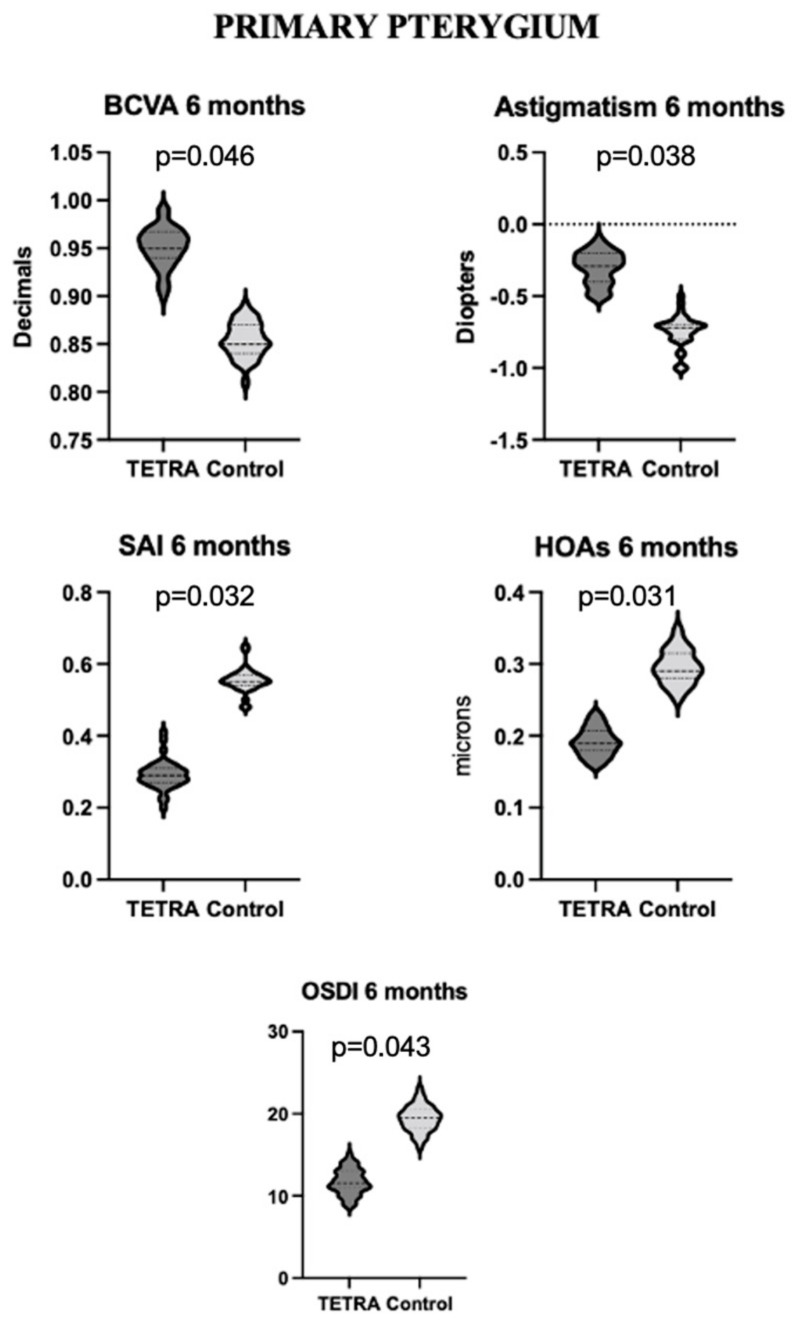
Violin plots of BCVA, astigmatism, SAI, HOAs, and OSDI score at 6-month follow-up in patients with primary pterygium from TETRA and control groups. Differences reported at 6 months remained stable at 1-year follow-up. Six-month timepoint can thus be regarded as the time of first appearance of definitive differences between treated and naïve patients. BCVA = best corrected visual acuity; HOAs = high-order aberrations; OSDI = ocular surface disease index; SAI = surface asymmetry index.

Primary pterygia manifested a significantly higher BCVA at 6-week (*p* = 0.039), 6-month (*p* = 0.046), and 1-year (*p* = 0.048) follow-up in the TETRA group. Moreover, they manifested no significant difference in spherical refractive error (*p* = 0.832) but significantly lower astigmatism at 6-week (*p* = 0.023) and 6-month (*p* = 0.038) follow-up. The LSA and IC did not differ significantly between the two groups in primary pterygium patients (*p* = 0.711 and *p* = 0.694). By contrast, the SAI was significantly different at 6-week (*p* = 0.045), 6-month (*p* = 0.032), and 1-year (*p* = 0.027) follow-up. Similarly, the corneal total RMS was lower in the TETRA group at 6 weeks and 1 year (respectively, *p* = 0.041 and *p* = 0.022), and HOAs were lower in the TETRA group at 6-week, 6-month, and 1-year follow-up (respectively, *p* = 0.044, *p* = 0.031, and *p* = 0.011). The OSDI score was also lower in the TETRA group at each follow-up timepoint (respectively, *p* = 0.037, *p* = 0.043, and *p* = 0.049). The main differences between the TETRA and control groups at 6-month follow-up are illustrated in Figure 2. 

#### 3.3.2. Recurrent Pterygium

As concerns recurrent pterygia, there were no significant differences in terms of age, sex composition, ethnicity, sun exposure, and laterality between patients in the TETRA and control groups. Moreover, no significant difference in terms of BCVA, spherical refractive error, cylindrical refractive error, LSA, IC, SAI, corneal total RMS, and OSDI score were detected between the two groups at baseline (*p* < 0.05). The rate of recurrence was significantly lower in the TETRA group (*p* < 0.001). In fact, in the TETRA group, one eye developed a recurrence (1.7 mm) at the 1-year timepoint (1/24 (4.16%)). In the control group, 3/19 (15.8%) eyes developed a recurrence: one (1.5 mm) at the 3-month timepoint, and two (2.2 mm and 2.5 mm) at the 6-month timepoint.

**Figure 3:**
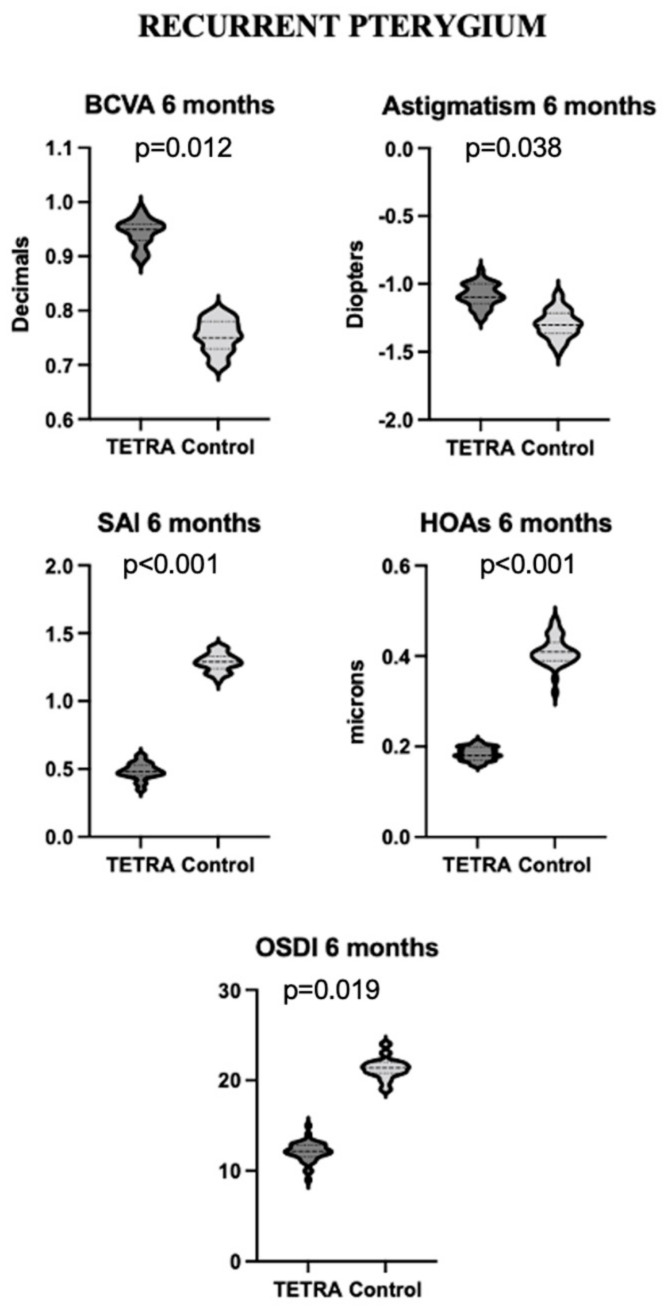
Violin plots of BCVA, astigmatism, SAI, HOAs, and OSDI score at 6-month follow-up in patients with recurrent pterygium from TETRA and control groups. Differences reported at 6 months remained stable at 1-year follow-up. Six-month timepoint can thus be regarded as the time of first appearance of definitive differences between treated and naïve patients. BCVA = best corrected visual acuity; OAs = high-order aberrations; OSDI = ocular surface disease index; SAI = surface asymmetry index.

Patients in the TETRA group manifested a significantly higher BCVA at 6-week (*p* = 0.036), 6-month (*p* = 0.012), and 1-year (*p* < 0.001) follow-up compared to the control group. Significantly lower astigmatism was detected in the TETRA group at 6-week (*p* = 0.032), 6-month (*p* = 0.038), and 1-year (*p* = 0.039) follow-up. The SAI was significantly different at 6-week (*p* < 0.001), 6-month (*p* < 0.001), and 1-year (*p* < 0.001) follow-up between the two groups. Similarly, the corneal total RMS was lower in the TETRA group at 6-week, 6-month, and 1-year follow-up (respectively, *p* = 0.001, *p* = 0.002 and *p* = 0.001). HOAs were lower in the TETRA group at 6-week, 6-month, and 1-year follow-up (respectively, *p* = 0.009, *p* < 0.001, and *p* < 0.001). The OSDI score was also lower in the TETRA group at each follow-up timepoint (respectively, *p* = 0.017, *p* = 0.019, and *p* = 0.009). The main differences between the TETRA and control groups at 6-month follow-up in recurrent pterygia are illustrated in Figure 3.

## 4. Discussion

Pterygium affects around 6% of people over the age of 40 in European countries [43], with older age, male gender, outdoor occupations, and Hispanic ethnicity being important risk factors [44]. Its pathogenesis occurs in two stages, including the initial and progressive disruption of the limbal corneal–conjunctival epithelial barrier and subsequent progressive active conjunctivalization of the cornea characterized by extensive cellular proliferation, inflammation, connective tissue remodeling, and angiogenesis. The pathologic tissue is hypothesized to arise from UV-altered limbal stem cells [45]. Reactive oxygen species induced by UV light also cause the breakdown of the extracellular matrix and alter the synthesis of collagen and elastin [46]. Lastly, oxidative stress is shown to promote metalloprotease-2 (MMP-2) activity and reduce tissue inhibitor of metalloproteases-1 (TIMP-1), which are events that significantly influence the growth of the pterygium [47]. In fact, the histologic features of pterygium, which include proliferation, cell migration, inflammatory infiltrates, and extracellular matrix remodeling, are explained by the actions of multiple proinflammatory cytokines, growth factors, and metalloproteases (MMPs), where their presence is consistent with their involvement in the corneal wound-healing cascade. [48] MMPs are a family of zinc-dependent endopeptidases capable of denaturing most components of the extracellular matrix. MMPs are regulated at multiple levels, including inhibition, which is provided by tissue inhibitors of MMPs (TIMPs) [49]. Maintenance of the equilibrium between MMPs and TIMPs is essential, and any disturbance in the balance is a critical determinant of proteolysis and tissue invasion [50]. Altered limbal basal epithelial cells (pterygium cells) produce abnormally elevated MMP-1, MMP-2, and MMP-9, which are the main factors responsible for the invasion and dissolution of Bowman’s layer in this disease. In fact, MMP-2 is specifically overexpressed in the epithelium and MMP-9 is abundant in pterygium vascular endothelium and inflammatory cells [47,49,51,52]. This pathogenetic background induced us to retrospectively investigate the rate of recurrence of pterygium after surgical excision and conjunctival autograft apposition in patients under chronic treatment with LD-SD for refractory blepharitis. In fact, LD-SD has been shown to decrease the production of IL-1 and MMPs by about 70% in corneal epithelial cells [53]. LD-SD also exerts a strong anti-inflammatory effect via the inhibition of TNF alfa production and MAP-K activation and attenuating the local effects of VEGF [23,27,29,54]. For these reasons, LD-SD has been approved and tested for the treatment of several ocular surface and corneal inflammatory diseases [22,31,32,33]. According to our retrospective analysis, recurrences of primary pterygium were significantly less prevalent in patients under treatment with LD-SD, both in the primary pterygium and the recurrent pterygium cohorts. In fact, only one patient in the primary pterygium cohort (2.77%) presented a recurrence after 1 year from excision compared to three patients (9.09%) in the control group. Despite being limited by the retrospective nature of this study, the recurrence rate in the TETRA group also testifies an improvement in comparison to the literature-reported rate of 1-year recurrence in both untreated patients and patients treated with topical cyclosporine [55,56], topical steroids alone [57], topical bevacizumab alone [13], artificial tears alone [58], a combination of topical dexamethasone and hydroxy-propyl-methyl-cellulose artificial tears [59], and topical autologous serum drops [58]. Even more surprisingly, patients from the recurrent pterygium cohort in the TETRA group manifested only one case of recurrence after 1 year (4.16%), which is considerably lower compared to the usual rate in untreated patients and patients treated with other topical methods [60]. By contrast, a rate of recurrence similar to the one in our study was reported in patients receiving topical therapy with a combination of corticosteroids, antibiotic therapy, and topical bevacizumab (2.4%) [61]. Nevertheless, the low compliance related to a chronic treatment with three different eyedrops along with the local side effects of both bevacizumab and corticosteroid therapy should be taken into account. Su et al. [29] reported that a combination of topical doxycycline temperature-sensitive hydrogel and topical bevacizumab effectively inhibited corneal neovascularization and corneal wound healing in an animal model of corneal burn healing. In particular, they postulated that topical doxycycline enhances the inhibitory effects of bevacizumab on corneal neovascularization while preventing its side effects on corneal wound healing, possibly by inhibiting the expression and activity of MMPs, the expression of VEGF, phosphorylated VEGF receptor 1 and 2, and the production of iNOS and IL-1β. In this perspective, it would be interesting to test the efficacy of a combined treatment with topical low-dose doxycycline and topical bevacizumab in the prevention of pterygium recurrence. It is important to acknowledge that the effect of LD-SD on the prevention of pterygium recurrence observed in our study is certainly influenced by the improvement of blepharitis/DED in these patients due to LD-SD itself. In fact, since the persistence of blepharitis/DED in the postoperative period is itself a predisposing factor for pterygium recurrence, the improvement of this condition due to LD-SD treatment in our cohort acts as an intrinsic confounder in the evaluation of the effect of LD-SD on pterygium alone.

As concerns secondary outcomes, BCVA at the 1-year postoperative follow-up improved significantly from the baseline in both groups, both in primary and recurrent pterygia. Nevertheless, BCVA at 6-week, 6-month, and 1-year follow-up was significantly higher in the TETRA group compared to the control group in both primary and recurrent pterygia. In addition to the beneficial effect of the pterygium removal, this difference could be explained by the improvement in ocular surface regularity induced by the restoration of the tear film distribution and the reduction in HOAs in LD-SD-treated patients. In fact, the air/tear interface contributes 70% of the vergence (focusing power) in the eye and, because of this, even minor variations in its shape can produce a significant visual deficit [62]. Therapy with LD-SD may potentially promote these indirect mechanisms since its anti-inflammatory effect on the ocular surface facilitates re-epithelization regularity and improves tear film quality. As a confirmation, LD-SD-treated patients also experienced a reduction in the SAI at all considered follow-ups, which was not present in the control group. At the same time, the total corneal aberrations decreased in primary and recurrent pterygium from the TETRA group after surgery, and recurrent pterygia also experienced a significant reduction in HOAs. Moreover, HOAs significantly differed between the TETRA and control groups at all considered follow-up examinations. Notably, even though the most important changes occurred between the baseline and 6-week evaluations, topographic and aberrometric data continued to improve over the course of the first postoperative year despite therapy with LD-SD having not endured the whole follow-up period. Interestingly, Yilmaz et al. [63] did not report significant changes in topographical corneal surface regularity after pterygium excision with neither the bare sclera technique, excision with adjuvant mitomycin C, excision with limbal–conjunctival autograft, nor excision with conjunctival autograft. Lastly, primary pterygium and recurrent pterygium patients from the TETRA group both reported a reduction in dry-eye symptoms, testified by a decrease in the OSDI score from pathologic levels in the preoperative period to normal levels after surgery. By contrast, a reduction in the OSDI score was reported only for primary pterygia in the control group. Coherently, LD-SD alone has been reported to significantly increase both tear break up time and Schirmer test performances [37]. Unfortunately, an MGD severity assessment was not available for all patients in our population due to the retrospective nature of this study. Nevertheless, the OSDI score at baseline was not significantly different between groups and it was significantly lower in postoperative assessments in patients treated with LD-SD. This could be the manifestation of a parallel beneficial effect on MGD severity in these patients. Once again, the therapeutic effect of LD-SD on DED/blepharitis in our cohort is likely to be responsible for the improvement in the OSDI score. To sum up, the beneficial effect of the use of LD-SD detected in our study could be attributable to two different mechanisms that are not easily separable: on one side, the possible growth inhibitory mechanism at the level of the lesion (as described above), and on the other side, the positive effect on collateral factors predisposing to recurrence and dry-eye symptoms (such as DED and blepharitis themselves), which act as confounders in our case. In conclusion, based on our findings, it is reasonable to encourage treatment with LD-SD in patients affected by chronic refractory blepharitis that are planning to undergo surgical removal of the pterygium and that show a good tolerance profile. The main limitation of this study is its retrospective nature, which limited the standardization of procedures and treatment. The lack of a placebo-controlled group should also be acknowledged as a limitation to our findings. Moreover, it has to be considered that the application of this therapeutic approach might be limited by systemic side effects inherent with the oral treatment, including potential teratogenic effects, gastrointestinal disturbance, and skin hyperpigmentation [37]. Rare cases of intracranial hypertension, anosmia, and hypoglycemia have also been reported in the literature (<0.01%) [64]. Gastrointestinal adverse effects (especially esophagitis and gastritis) are the most common, with a prevalence of around 6% [65]. In patients assuming SD for more than one month, their prevalence is higher for doses > 200 mg per day of SD and in patients older than 50 years. In our population, three patients (5.0%) experienced gastritis or esophagitis and only one patient suspended treatment due to adverse effects after a treatment period of 6 months. No cases of skin hyperpigmentation, intracranial hypertension, anosmia, or hypoglycemia were reported. None of our female patients were pregnant. It should be reminded that LD-SD is strictly contraindicated during pregnancy. Nonetheless, the promising results of our investigation and the rationale of the treatment should prompt further prospective studies and randomized controlled trials to confirm our findings, possibly also including a parallel assessment of MGD and demodicosis and their influence on treatment efficacy.

## Figures and Tables

**Table 1:** Description of characteristics and results of one-way ANCOVA for repeated measures in both primary and recurrent pterygia in TETRA group. BCVA = best corrected visual acuity; D = diopters; HOAs = high-order aberrations; IC = symmetry index of curvature; OSDI = ocular surface disease index; RE = right eye; RMS = root mean square; SAI = surface asymmetry index. * = statistically significant values.

TETRA Group					
Primary	Baseline	6 Weeks	6 Months	1 Year	*p*
Age	45.69 ± 14.27				
Male sex	16/36 (44.4%)				
Ethnicity	Caucasian = 22/36 (61.1%) Hispanic = 14/36 (38.9%)				
Sun exposure > 5 h/day	16/36 (44.4%)				
Eye	RE = 18/36 (50%)				
BCVA (Decimals)	0.79 ± 0.11	0.92 ± 0.06	0.96 ± 0.038	0.96 ± 0.038	0.037 *
Sphere (D)	1.09 ± 1.34	1.08 ± 1.94	1.08 ± 1.94	1.08 ± 1.94	0.851
Cylinder (D)	−1.17 ± 0.95	−0.25 ± 0.73	−0.25 ± 0.73	−0.25 ± 0.73	0.021 *
SAI	0.67 ± 0.49	0.41 ± 0.29	0.29 ± 0.25	0.24 ± 0.21	0.042 *
LSA	0.95 ± 0.72	0.85 ± 0.71	0.78 ± 0.50	0.66 ± 0.43	0.063
IC	0.18 ± 0.12	0.16 ± 0.11	0.15 ± 0.09	0.14 ± 0.10	0.649
RMS tot	0.59 ± 0.48	0.47 ± 0.38	0.37 ± 0.29	0.24 ± 0.08	0.039 *
HOAs	0.32 ± 0.27	0.20 ± 0.15	0.19 ± 0.15	0.13 ± 0.11	0.058
OSDI	26.85 ± 8.42	14.07 ± 6.15	11.50 ± 4.57	10.37 ± 3.66	0.025 *
Recurrence	1/36 (2.77%)				
**Recurrent**	**Baseline**	**6 weeks**	**6 months**	**1 year**	***p***
Age	50.31 ± 9.62				
Male sex	10/24 (41.67%)				
Ethnicity	Caucasian = 4/24 (16.67%) Hispanic = 20/24 (83.33%)				
Sun exposure > 5 h/day	4/24 (16.67%)				
Eye	RE = 14/24 (58.33%)				
BCVA	0.56 ± 0.19	0.84 ± 0.058	0.95 ± 0.04	0.94 ± 0.06	<0.001 *
Sphere	1.67 ± 1.02	1.55 ± 1.13	1.71 ± 1.17	1.70 ± 1.12	0.714
Cyl	−2.53 ± 0.83	−1.18 ± 0.37	−1.10 ± 0.22	−1.15 ± 0.33	0.002 *
SAI	1.31 ± 0.82	0.59 ± 0.28	0.47 ± 0.23	0.33 ± 0.26	0.004 *
LSA	1.35 ± 1.08	0.88 ± 0.38	0.76 ± 0.37	0.64 ± 0.26	0.021 *
IC	0.45 ± 0.16	0.25 ± 0.15	0.23 ± 0.12	0.20 ± 0.12	0.043 *
RMS tot	1.11 ± 0.88	0.70 ± 0.38	0.60 ± 0.28	0.51 ± 0.25	0.036 *
HOAs	0.49 ± 0.56	0.31 ± 0.41	0.18 ± 0.14	0.15 ± 0.07	0.041 *
OSDI	24.0 ± 8.91	15.33 ± 6.38	12.17 ± 3.38	10.76 ± 2.73	0.005 *
Recurrence	1/24 (4.16%)				

**Table 2:** Description of characteristics and results of one-way ANCOVA for repeated measures in both primary and recurrent pterygia in control group. BCVA = best corrected visual acuity; D = diopters; HOAs = high-order aberrations; IC = symmetry index of curvature; OSDI = ocular surface disease index; RE = right eye; RMS = root mean square; SAI = surface asymmetry index. * = statistically significant values.

CONTROL Group					
Primary	Baseline	6 Weeks	6 Months	1 Year	*p*
Age	41.39 ± 13.17				
Male sex	15/33 (45.5%)				
Ethnicity	Caucasian = 21/33 (63.6%) Hispanic = 12/33 (34.6%)				
Sun exposure > 5 h/day	13/33 (39.4%)				
Eye	RE = 16/33 (48.5%)				
BCVA (Decimals)	0.75 ± 0.10	0.81 ± 0.09	0.86 ± 0.04	0.87 ± 0.05	0.045 *
Sphere (D)	1.51 ± 0.95	1.39 ± 1.04	1.38 ± 1.06	1.39 ± 1.07	0.463
Cylinder (D)	−1.27 ± 0.82	−1.15 ± 0.71	−0.75 ± 0.53	−0.25 ± 0.73	0.028 *
SAI	0.59 ± 0.22	0.57 ± 0.27	0.55 ± 0.36	0.58 ± 0.21	0.561
LSA	0.93 ± 0.56	0.89 ± 0.64	0.86 ± 0.61	0.84 ± 0.63	0.499
IC	0.17 ± 0.11	0.15 ± 0.10	0.15 ± 0.09	0.16 ± 0.11	0.672
RMS tot	0.61 ± 0.37	0.57 ± 0.38	0.41 ± 0.26	0.39 ± 0.13	0.046 *
HOAs	0.33 ± 0.21	0.28 ± 0.17	0.29 ± 0.15	0.28 ± 0.13	0.112
OSDI	25.32 ± 6.81	22.97 ± 7.35	19.58 ± 6.17	17.11 ± 5.26	0.046 *
Recurrence	3/33 (9.09%)				
**Recurrent**	**Baseline**	**6 weeks**	**6 months**	**1 year**	***p***
Age	52.41 ± 7.11				
Male Sex	8/19 (42.11%)				
Ethnicity	Caucasian = 4/19 (21.05%) Hispanic = 15/19 (78.9%)				
Sun exposure > 5 h/day	4/19 (21.05%)				
Eye	RE = 9/19 (47.36%)				
BCVA (decimals)	0.59 ± 0.13	0.72 ± 0.09	0.75 ± 0.12	0.81 ± 0.09	0.027 *
Sphere (D)	1.08 ± 0.75	1.21± 0.69	1.31 ± 0.54	1.10 ± 0.74	0.438
Cylinder (D)	−2.27 ± 1.03	−1.62 ± 0.41	−1.30 ± 0.34	−1.35 ± 0.33	0.029 *
SAI	1.45 ± 0.73	1.29 ± 0.68	1.27 ± 0.53	1.33 ± 0.61	0.516
LSA	1.25 ± 0.17	1.08 ± 0.22	0.96 ± 0.34	1.04 ± 0.36	0.213
IC	0.41 ± 0.11	0.35 ± 0.12	0.37 ± 0.11	0.38 ± 0.12	0.395
RMS tot	1.27 ± 0.53	1.00 ± 0.48	0.92 ± 0.31	0.81 ± 0.29	0.041 *
HOAs	0.52 ± 0.31	0.46 ± 0.39	0.41 ± 0.26	0.42 ± 0.23	0.225
OSDI	27.33 ± 6.11	25.09 ± 6.35	21.28 ± 5.88	20.93 ± 5.83	0.072 *
Recurrence	3/19 (15.79%)				

**Table 3:** Comparison of other relevant characteristics in the two study groups. DED = dry-eye disease.

	TETRA Group (60 Eyes)	CONTROL Group (52 Eyes)	*p*
Age (years)	47.6 ± 7.22	46.9 ± 6.03	0.890
Sex	26/60 (43.3%)	23/52 (44.2%)	0.923
Hispanic ethnicity	34/60 (56.7%)	27/52 (51.9%)	0.615
Outdoor working and/or sun exposure > 5 h/day	20/60 (33.3%)	17/52 (32.7%)	0.942
Preoperative DED	Mild = 15/60 (25.0%) Moderate = 11/60 (18.3%) Severe = 4/60 (6.7%)	Mild = 11/52 (21.2%) Moderate = 10/52 (19.2%) Severe = 3/52 (5.7%)	0.959
Preoperative treatment with artificial tears	37/60 (61.6%)	30/52 (57.7%)	0.668
Preoperative inflammation/pterygium congestion	8/60 (13.3%)	10/52 (19.2%)	0.396
Pterygium largest diameter (mm)	2.8 ± 1.3	2.7 ± 1.7	0.299
Visual axis involvement	22/60 (36.7%)	18/52 (34.6%)	0.821
Chronic pterygium	17/60 (28.3%)	19/52 (36.5%)	0.353
Recurrent pterygium	24/60 (40.0%)	19/52 (36.5%)	0.707
Surgical technique	Autograft = 22/60 (36.7%) Transposition = 38/60 (63.3%)	Autograft = 17/52 (32.7%) Transposition = 35/52 (67.3%)	0.659
Postoperative inflammatory episodes (number of episodes during follow-up)	0.4 ± 0.6	0.8 ± 1.3	0.065

## Data Availability

Data are available upon reasonable request to the corresponding author.
